# Left circumflex artery occlusion during accessory pathway radiofrequency ablation: Be ready for the worst

**DOI:** 10.1016/j.hrcr.2025.03.023

**Published:** 2025-04-03

**Authors:** Hamza A. Abdul-Hafez, Reem A. Abu-Alrub, Fateh Awwad, Ibraheem Marie, Abdul-Rahman R. Abdel-Karim

**Affiliations:** 1 Department of Medicine, Faculty of Medicine and Health Sciences, An-Najah National University, Nablus, Palestine; 2 Department of Cardiology, An-Najah National University Hospital, An-Najah National University, Nablus, Palestine; 3 Department of Interventional Cardiology, An-Najah National University Hospital, Nablus, Palestine

**Keywords:** Radiofrequency ablation, Arrhythmia, Accessory pathway, Wolff-Parkinson-White (WPW), Left circumflex artery occlusion, Percutaneous coronary intervention

**Key Teaching Points**

• Electrophysiologists need to be extra vigilant and expect coronary complications such as total arterial occlusion, especially to the left circumflex artery during coronary sinus accessory pathway ablations.
• Operators need to closely monitor for new and significant ST segment changes on the monitor during similar cases and to take that seriously and perform coronary angiography to diagnose and treat similar complications.
• We recommend avoiding or limiting sedation in such cases not to obscure anginal symptoms. Consider doing preoperative coronary angiography and CS venogram before to help better guide procedure and predict possible similar complications.
• We recommend having intracoronary imaging software such as intravascular ultrasound (IVUS) or optical coherence tomography in the EP catheterization laboratory to help further understand the pathophysiology and better treat such complications.
• Further studies are needed to compare different therapeutic options, such as systemic steroids or thrombectomy or watchful waiting and monitoring compared with conventional balloon angioplasty or stenting in such complications, especially among young, otherwise healthy patients. To show significant differences, a large number of patients would be needed, which we are not sure is possible knowing it is a relatively rare complication.

## Introduction

Wolff-Parkinson-White (WPW) syndrome is a tachyarrhythmia conduction disorder characterized by presence of an accessory pathway bypassing the atrioventricular (AV) node leading to tachyarrhythmia and rarely death. Radiofrequency (RF) ablation is considered the treatment of choice for such accessory pathway especially when associated with WPW syndrome. Success rates are usually high,^1^^,^^2^ and in less than 5% of cases, complications such as bleeding, vascular injury, or coronary stenosis thought to be attributable to thermal injury and tissue edema can occur.

Coronary artery stenosis or occlusion as a complication of RF ablation is exceedingly rare, especially in young patients without significant comorbidities.^3^ A limited number of reported cases describing coronary artery occlusion after ablation of WPW syndrome accessory pathway were found (Table 1).

**Table 1:** Literature review of similar cases

No.	1st author	Year of publication	Gender/age	Occluded artery	Indication of ablation	Site of ablation	Management of postablation occlusion	Outcome of management
1.	Makimoto et al^14^	2014	M/66	LCx	Atrial tachycardia	CS (around mitral annulus)	Drug-eluting stent	Excellent angiographic result
2.	Yalin et al^11^	2012	M/56	LMCA	WPW syndrome	Left anterolateral AP	Bare metal stent	Improved , discharged completely asymptomatic after 7 days
3.	Wong et al^8^	2010	M/62	LCx	Symptomatic persistent atrial tachycardia refractory to medical treatment	Mitral isthmus	Balloon angioplasty, stent placement	Uneventful recovery
4.	Yildiz et al^15^	2014	M/38	LAD	WPW syndrome	Left free wall AP	Thrombectomy catheter, stent placement	Chest pain decreased and ST-segment elevation was resolved
5.	Obeyesekere et al^16^	2011	M/35	RCA	Preexcitation consistent with accessory pathway	Posteroseptal AP	Coronary angioplasty with stent placement	Management succeeded
6.	Hardy et al^17^	2022	M/18	RCA	Recurrent atrioventricular reentrant tachycardia	Right posteroseptal AP	Coronary angioplasty with stent placement	Complete resolution of pain
7.	Khanal et al^18^	1999	M/12	RCA	WPW syndrome	Right posteroseptal AP	Balloon Angioplasty, stent placement	Discharged home the following day in good condition
8.	Yildiz et al^10^	2020	M/5	LCx	WPW Syndrome	Left lateral AP	Balloon angioplasty, systemic steroid to resolve myocardial edema	Patient improved and became hemodynamically stable
9.	Wong et al^9^	2011	50–70	LCx	Atrial fibrillation	Mitral isthmus	Intracoronary glycerine trinitrate boluses	Narrowing resolved

AP = accessory pathway; CS = coronary sinus; LAD = left anterior descending artery; LCx = left circumflex artery; LMCA = left main coronary artery; M = male; RCA = right coronary artery; WPW = Wolff-Parkinson-White.

In this case report, we present a young patient who developed inferior ST segment elevation observed on the monitor during his ablation procedure, with minimal associated symptoms such as chest pain or dyspnea. He was hemodynamically stable. Because of the new electrocardiogram (ECG) changes, the interventional cardiologist on call was called in and an emergent coronary angiography was performed, which confirmed the suspected occluded left circumflex (LCX) artery likely caused by an external thermal injury and compression. Percutaneous coronary intervention (PCI) was performed, and the patient was discharged in stable condition the following day.

## Case presentation

A 24-year-old man, smoker but otherwise healthy, was diagnosed with WPW syndrome after having recurrent palpitations, dizziness, and sweating. He was initially evaluated and diagnosed at an outside hospital. He was then admitted to our tertiary hospital for a planned RF ablation of his accessory pathway.

**Figure 1:**
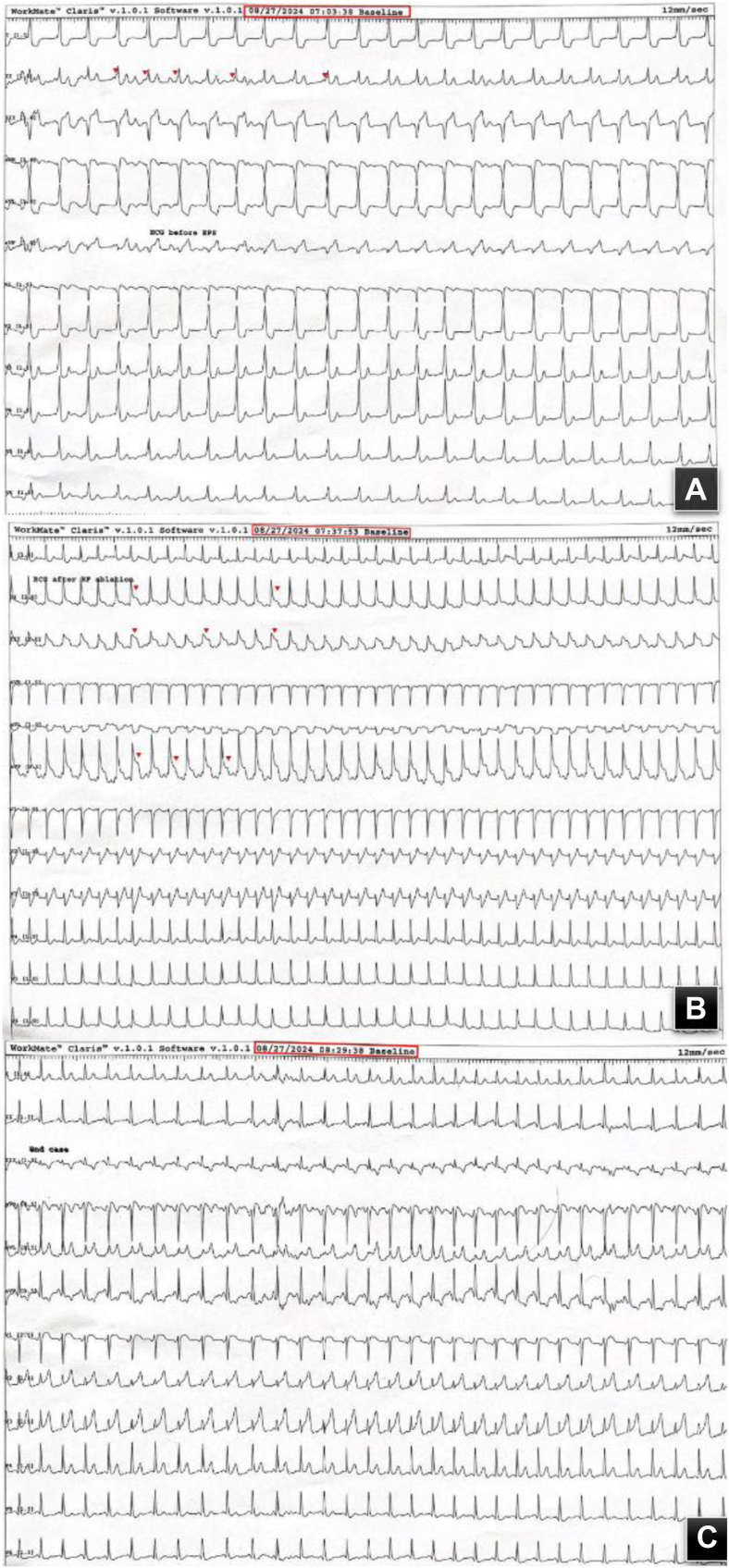
**A:** Baseline 12-lead ECG with pre-excitation noted (*red arrowheads*). **B:** 12-leads ECG postablation showing new inferior ST segment elevation (*red arrowheads*). **C:** Postablation 12-lead ECG showing resolution of pre-excitation and resolution ST segment elevation. ECG = electrocardiogram.

On admission to our hospital, the patient had normal vital signs. His ECG showed a normal sinus rhythm, short PR interval, and the presence of a Delta wave (Figure 1A). The patient was in good overall health, with no complaints of chest pain, dizziness, or sweating. Physical examination was unremarkable, and laboratory findings were within normal limits.

**Figure 2:**
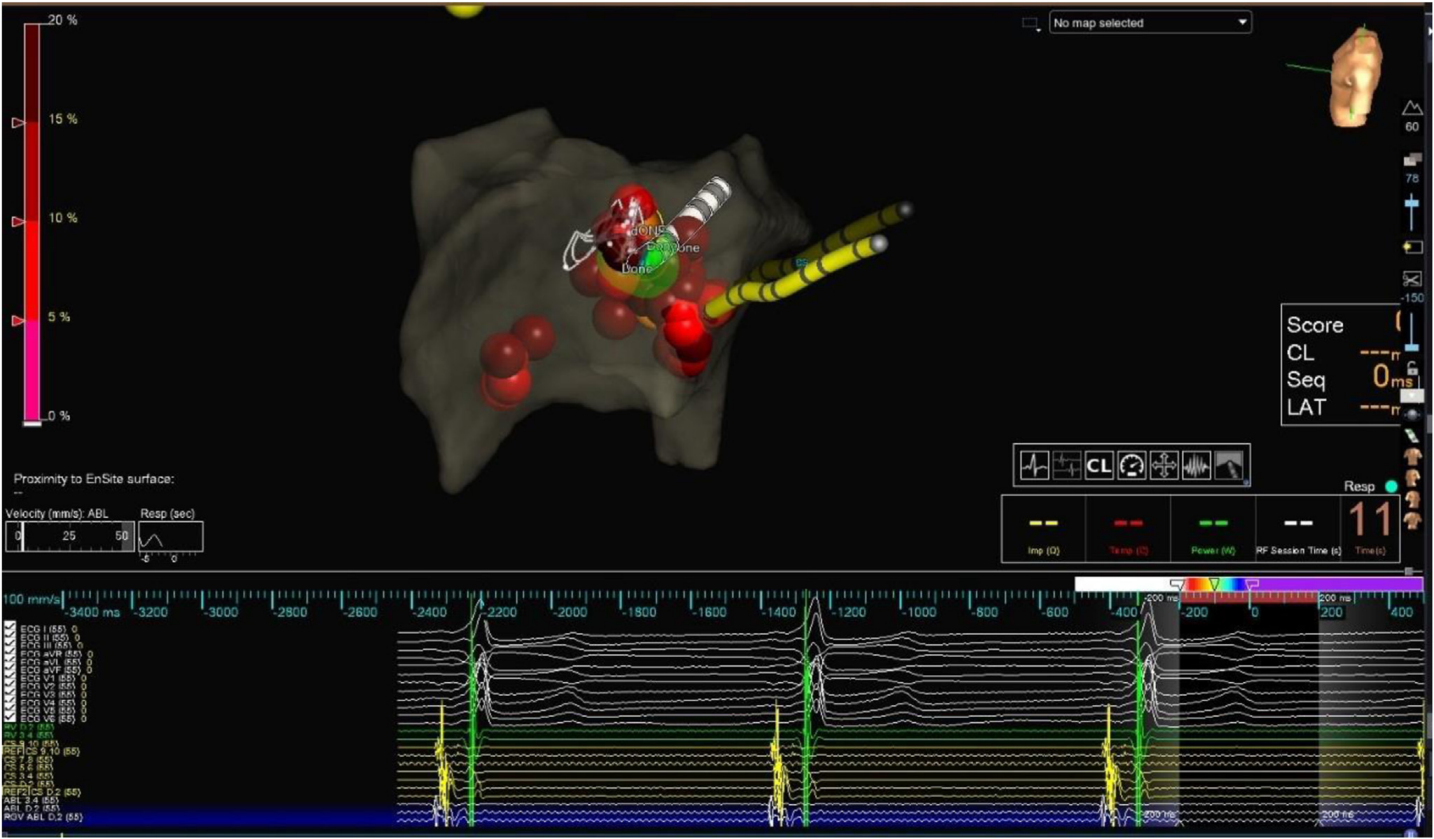
3D mapping showing ablation location foci.

After obtaining informed consent, an electrophysiologic procedure was performed under conscious sedation, using right femoral vein access. Manifest pre-excitation was observed (Figure 1A), characterized by a negative delta wave in leads V1, III, AVF, and a positive delta wave in leads I, AVL, V2-6. Using 3D electrophysiology mapping, the accessory pathway was localized in the right posteroseptal area and close to the coronary sinus (CS) ostium (Figure 2). Initially after first RF application, preexcitation was eliminated; however, early recurrence of preexcitation was noticed, requiring multiple RF ablations. During the final ablation, which was performed inside proximal CS close to the orifice of the middle cardiac vein (The Navex X 3D Mapping and Ablation System from Abbott Medical was used with an irrigated, flexible ablation catheter (Bi-D, D-F) at 35 W power, 42 °C temperature, and 100-ohm impedance), a new ST segment elevation (STE) without preexcitation was observed in the inferior leads (Figure 1B).

**Figure 3:**
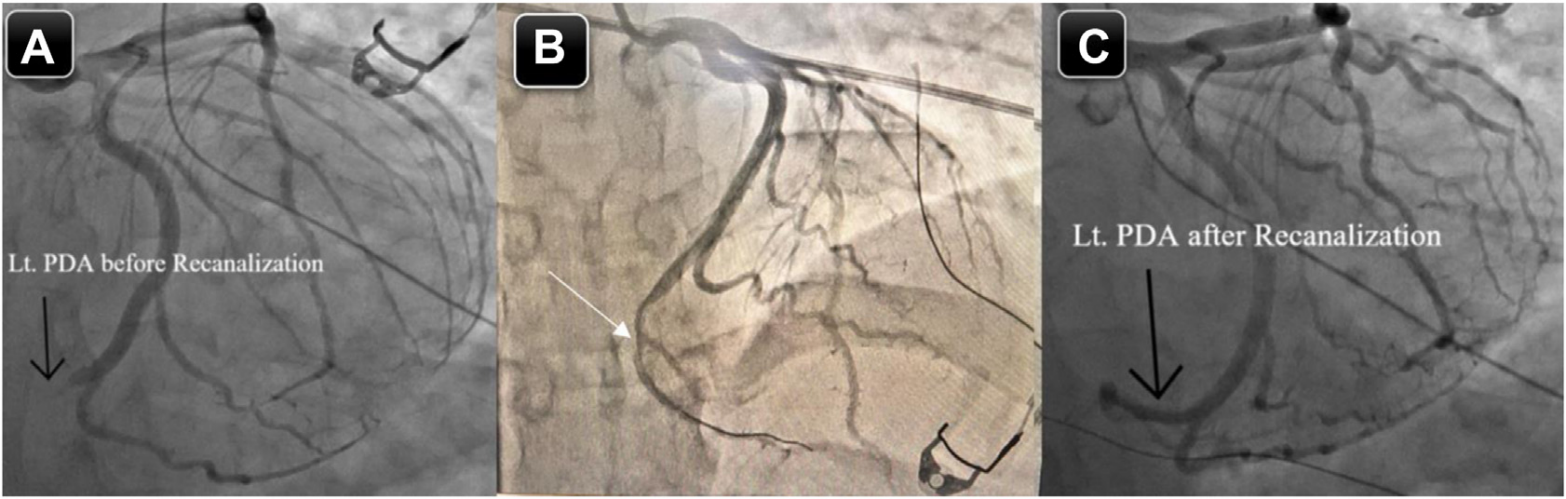
**A:** Coronary angiography showing complete occlusion of the distal LCX artery**. B:** Angiography post balloon angioplasty to the LCX artery showing residual significant stenosis (*white arrow*)**. C:** Coronary angiography post successful PCI using a DES to the distal LCX artery. DES = drug eluting stent; LCX = left circumflex; PCI = percutaneous coronary intervention.

The application was immediately stopped. Intravenous nitroglycerin was given, with improvement in his ST segment elevation. It is worth mentioning that the patient denied any chest pain or discomfort; an emergent coronary angiography was performed while the patient was still on the EP catheterization table, which showed an occluded distal dominant left circumflex artery (Figure 3A). As mentioned earlier, nitroglycerin was used, then passage of a coronary guidewire was done, despite the fact that no restoration of flow was achieved and thus balloon angioplasty had to be used to restore flow (Supplementary Videos 1, 2, and 3). After that there was significant residual luminal narrowing (Figure 3B), and PCI using a drug-eluting stent had to be performed to the distal left circumflex artery, with an excellent angiographic result (Figure 3C) and no complications. Complete resolution of his STE was noted (Figure 1C). Intravascular imaging was not done because of the emergent type of this case as well as the fact that we do not have the software already installed in the EP catheterization laboratory, where this case was done.

**Figure 4:**
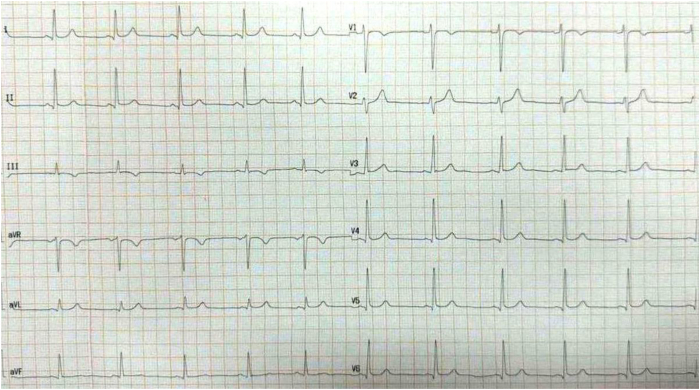
12-lead electrocardiogram on follow-up showed normal PR interval without evidence of preexcitation.

On the following day, the patient was in good health with no symptoms. He was discharged home on dual antiplatelet therapy, high-intensity statin, and low-dose bisoprolol. The ECG showed a normal PR interval without evidence of preexcitation (Figure 4). A month later, our patient was seen in clinic for follow-up, with no recurrence of his palpitations and no chest pain. He remains adherent to his medical therapy, including dual antiplatelet therapy.

## Discussion

In this case, we reported a rare but life-threatening complication caused by RF ablation of WPW syndrome in a previously healthy young male. The complication was detected by closely monitoring the ECG during the procedure and was successfully managed using LCX artery PCI, with an excellent outcome.

Although RF ablation is the preferred and highly effective therapy for accessory pathway tachyarrhythmias,^1^^,^^2^ it can lead to various complications, including vascular injury, cardiac tamponade, heart block, stroke, and retroperitoneal bleeding.^3^ In extremely rare cases, as shown in Table 1, ST segment elevation myocardial infarction can happen as well. The exact mechanism of injury is not completely clear, although transient thermal injury leading to coronary spasm or external compression and stenosis seems to be the primary mechanism, in addition to inflammatory delayed necrosis and intimal hyperplasia that result in late-onset stenosis.^4^ The incidence of coronary thermal injury during or immediately after ablation is exceptionally low, as low as 0.06% to 0.1% in adults.^5^ One of the main regrets in our case is not having an intracoronary imaging modality already installed in our EP catheterization laboratory. Performing intravascular coronary imaging such as intravascular ultrasound or optical coherence tomography in the future will help us know more about the exact mechanism of such a complication and probably how to better prevent and treat it.

**Figure 5:**
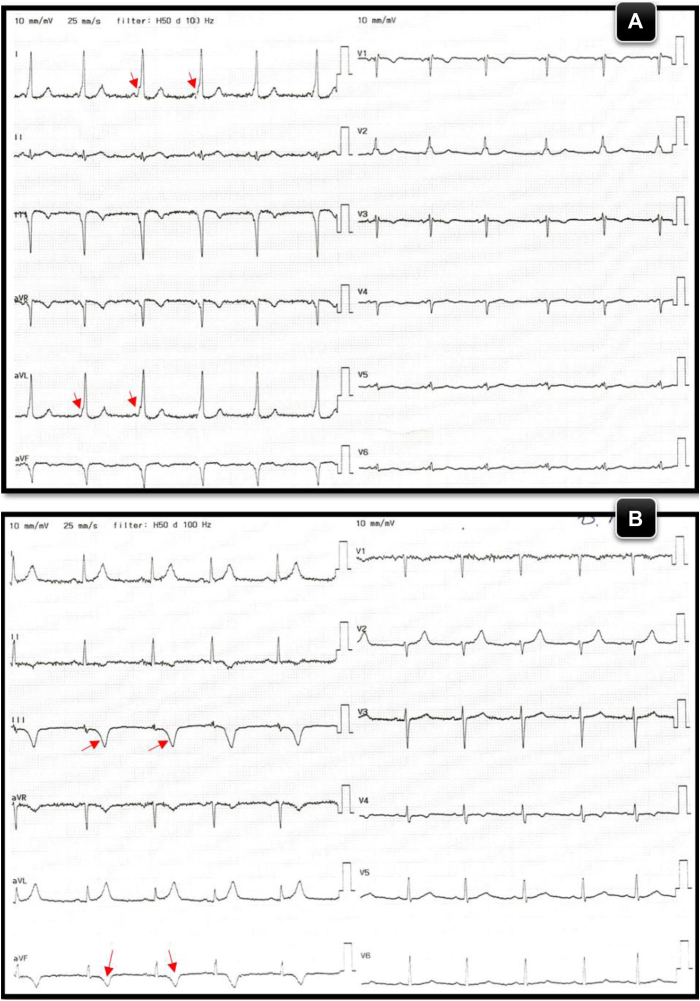
**A:** Baseline electrocardiogram of a similar patient with accessory pathway/Wolff-Parkinson-White syndrome (*arrows* showing Delta waves). **B:** Post successful accessory pathway ablation “cardiac memory” phenomena (*arrows* showing deep T wave inversion), with resolution of Delta waves in the same patient.

Post-ablation STE may arise from causes other than coronary artery injury. One such cause is cardiac memory, a phenomenon in which the ECG shows changes that mimic ischemic findings, without any evidence of actual stenosis or vasospasm.^6^ The patient with cardiac memory has no symptoms of myocardial infarction. However, in our case, despite absence of chest pain after STE, which could be masked by the conscious sedation our patient received, coronary angiography revealed a significant LCX occlusion that required balloon angioplasty and stenting. This underscores the importance of performing angiography after ablation, even in asymptomatic patients, when new STE is detected. Possibly the amount of ST segment elevation seen in our case is more significant than what typically is seen in “post ablation cardiac memory phenomena.” Cardiac memory is mainly manifested by T wave inversion and ST depression (Figure 5A, 5B) and rarely by impressive ST elevation such as what was seen in our case. Importantly, ST elevation or cardiac memory changes would be seen only after the disappearance of preexcitation. Thus, it is crucial to map the exact location of the accessory pathway before starting ablation (preferably with low power) and to stop ablation within seconds if there is no effect. Lack of ablation effect should prompt reevaluation and remapping to prevent several ablation attempts within the CS and to prevent unnoticed coronary artery damage caused by the presence of preexcitation, making the diagnosis of ECG changes very difficult.

The CS is becoming a more frequent targeted site of ablation in treating various arrhythmias, including accessory pathway, premature ventricular pathway, and mitral isthmus dependent flutter. Proximity of coronary artery poses the risk of STE and coronary artery stenosis. Ablation near the critical site, especially less than 3 to 5 mm from the coronary artery, increases the complication rate. In left-dominant patients, the LCX runs <2 mm from the proximal CS, whereas in right-dominant patients, the posterolateral branch of the right coronary artery (RCA) runs <2 mm from the CS.^7^

In the literature, most reported cases of LCX injury are associated with mitral isthmus ablation as treatment of atrial fibrillation or atrial flutter.^8^^,^^9^ Over the past 20 years, only 1 case of LCX occlusion associated with posteroseptal CS ablation has been reported.^10^ Yalin et al^11^ reported a WPW syndrome case of an older male patient complicated with left main coronary artery occlusion during ablation of the accessory pathway in the left anterolateral wall.

Compared with our case, which related to LCX occlusion during right posteroseptal accessory pathway ablation, a case reported by Isa et al^12^ described a young patient who experienced RCA occlusion 2 hours after successful WPW syndrome ablation. Typically, right posterolateral ablation-induced coronary occlusions involve the RCA, making LCX occlusion in our case particularly unusual.

In a study of 190 patients who underwent successful RF ablation for accessory pathways, Schläpfer et al^13^ reported a 4% incidence of acute complications, including valvular injury, femoral artery injury, and cerebral ischemic events. Notably, these complications were predominantly associated with left-sided accessory pathways. Our case highlights the potential for serious coronary complications even during right-sided ablations, emphasizing the need for careful intraoperative monitoring and postablation follow-up. It also opens the door to consider doing a preoperative assessment of the CS anatomy and its anatomic correlation to the coronary arteries, and if too close in proximity, to consider limiting the amount of thermal ablation or use cryoablation instead and have a low threshold to further investigate new and significant ST segment elevation even in asymptomatic patients.

We hypothesize that this complication can possibly be prevented by doing a coronary angiogram before ablation in the CS to assess for proximity of the coronary arteries, keeping relatively low power output and high irritation rate to avoid large lesions, or using cryoablation when too near coronary arteries. CS venography could also be performed, which may better guide manipulating the ablation catheter within the CS and its branches. Early detection of this complication is very important. We would suggest displaying 12 leads monitoring during ablation and terminating ablation when ST elevation is suspected. It is also important to differentiate between significant ST elevation as seen in our case and the benign well-known cardiac memory phenomena.

Also, other therapeutic modalities such as antiarrhythmic medications (IC group) should be considered when the risk of ablation such as AV block or coronary artery injury seems to be high.

## Conclusion

LCX artery occlusion occurred after ablation of the posteroseptal accessory pathway within the proximal CS. Operators should be aware of this serious complication. Urgent coronary angiography should be performed when new and significant ST elevation is noticed.

## Disclosures

All authors have no conflicts of interest to disclose.
