# Supplementary material for: Left circumflex artery occlusion during accessory pathway radiofrequency ablation: Be ready for the worst
Source: HeartRhythm Case Rep. 2025 Apr 3;11(6):580–7. doi: 10.1016/j.hrcr.2025.03.023 (PMC12184835; doi:10.1016/j.hrcr.2025.03.023)
Supplement: Supplementary Video 1 [file mmc1.docx]

**Videos Caption:**

**Video 1**: Left coronary angiography showing totally occluded distal LCX artery as well as late contrast filling of the coronary sinus.

**Video 2**: Left coronary angiography after advancing a guide wire in the LCX artery.

**Video 3**: Left coronary angiography after successful PCI to the distal LCX artery.
